# Sanitary Legislation

**Published:** 1857-07

**Authors:** 


### SANITARY LEGISLATION.

There has been a great deal of talk about Medical Reform, but all talk. A Bill from Sir George Grey intends to transform the Board of Health into a branch or Committee of Council ; which is the same, virtually, as presenting the Chiltern Hundreds to an honourable M.P. Mr. John Bull is consoled by the feeling that he can do without the Board, and will save by his loss as much as £4000 per annum. Sound logic, John, as based on recollections of the past. But oh, John Bull, John Bull, if you knew what could be done for sanitary science, and for your own health, to the tune of £4000 a year—if you knew what statistics of disease could be collected—what grand experiments could be made, such as would immortalize your England of this age—what facts and laws could be educed—what a promise of increasing national health and national wealth (the child of which is national happiness) could be secured, all for the said paltry sum which you for years have let placemen sink in their own funds—you would certainly abolish the present "how do you do, none the better for seeing you" concern, to replace it as certainly with something which you would be the better of seeing. The past Board of Health has in earnestness been a mistake, a squander, and almost a joke from the first. At its christening its parents mistook its name ; and now, in its brief decline, did it wish to leave the world with a clean breast, how well it might confessing say, that it has done those things which it ought not to have done, has left undone those things which it ought to have done, and that there has been no health in it. Let us be just ; a few reports of this Board have been worth the reading—a dozen perchance ; give line, and say two dozen. But compare these with the amount of individual and unpaid effort which has appeared in the same period ; and the science squalor of the Board becomes, by comparison, too pitiable even to be laughable.

Now, sanitarians all and true ; you who believe that in the knowledge of life laws, written by nature, is embodied a knowledge of the fundamental laws on which good government will ultimately rest—you who see in this day's sanitary labour a pass word for yourselves to next day honour—you who connect the physical and the mental life as in union inseparable, and as coming equally from the Supreme — you who work in hope, and in the assurance that whatever little you do is towards the regeneration of your country—you who know that England is still an unweeded garden, and requires vigorous cultivation—yes, all of you, work on, and in spite of Boards of Health, or no Boards of Health, the time will come when your science-goddess will reign.

### REPORT ON THE WATER SUPPLY OF THE METROPOLIS.

Dr. Hassall, in an official report on the drinking water of London, concludes thus :—

"It follows that the metropolis is still supplied with water containing considerable numbers of living vegetable and animal productions, and which are not present in the purer waters, as, for example, that supplied by the Plumstead, Woolwich, and Charlton Company. Contrasting these results with those obtained by the microscopical examinations of the waters supplied to the metropolis in 1854, that is, prior to the new act coming into operation, great improvement is undoubtedly manifest in the condition of the present supplies, as shown by the colour and taste of the waters, as well as by the diminished number of organic productions contained in them. It should be recollected, however, that the present examinations have been made in the midst of winter, that is, at the period most unfavourable to the development of animal and vegetable life ; the water now supplied to the metropolis is therefore in the purest state of which, under the present arrangements, it is susceptible. It is obviously proper that fresh examinations of the waters of the several companies should be made in the spring, summer, and autumn, in order that their conditions at those seasons might be determined. A very excellent test by which the quality of water may frequently be judged of, is the colour presented by it : this can only be judged of by viewing the water in bulk, that is, in a clear white glass bottle, or better still, in a porcelain dish, holding about a gallon, some distilled or pure spring water being placed in a corresponding dish for comparison. If the water thus viewed present any decided tinge or colouration, it in general is not pure. Inspected in this manner, the waters of the whole of the companies examined presented a very marked yellowish green colouration, which became more obvious as the water was concentrated by evaporation."

### REPORT ON DISINFECTION AND DEODORISATION.

A report on these subjects is by Mr. Lindsay Blyth, Analytical Chemist to the General Board of Health. Mr. Blyth states that the so-called disinfectants are of two classes :—" First, those which fix the organic matter in a form unfavourable to oxidation, and thus reduce to the utmost its tendency to undergo chemical changes—*Antiseptics ;* secondly, those which more or less rapidly break up the organic matter by promoting its oxidation and conversion into unputrefiable products—*Disenfectants.* The materials employed against those cases which are characterised by offensive smells are spoken of as *deodorisers ;* which word has thus come to be popularly used as equivalent to 'disinfectants.' This error should be guarded against, for there is no proof that the specific power of producing fever, small-pox, or cholera, is necessarily associated with any odorous matter. Sulphuretted or phosphoretted hydrogen may be smelt in places where there is no power of infection ; while in others, the mere absence or destruction of such odours, is not sufficient security against infection." He enumerates as these disinfectants fumes of nitric and nitrous acids, manganates and permanganates of potash and soda, fire, quicklime, and charcoal. For the disinfectants of occupied buildings, Mr. Blyth refers to chlorides of lime, soda, and zinc, and the manganates. Nitrous acid, chlorine, and sulphurous acid may be used in unoccupied buildings. For the disinfection of clothes he recommends exposure of them to heat, and in the appendix gives a brief account of Jeakes' and of Johnston's apparatus for this purpose. The appendix also embodies a report by Professor Hofmann on the manganates and permanganates of soda and potash, as applied by Mr. Condy of Battersea. The manganates give off their oxygen to bodies around them with great facility; hence their disinfecting power. Dr. Hofmann thus writes :—" The manganates and permanganates surpass in their deodorising and disinfecting powers most compounds which are usually employed for this purpose. Metallic salts, such as the compounds of lead, iron, zinc, etc., act extremely well, if the odour to be removed arise from sulphuretted hydrogen and ammonia, or substances analogous to the latter; when a metallic sulphide and a salt of a metal ammonium is formed. But, frequently, the odour belongs to substances of a different class, which are fixed by neither of the constituents of the metallic salt. The odour of the water, which in my experiments yielded perfectly to the action of the manganates, was scarcely altered by the use of very considerable quantities of the usual metallic salts. Moreover, the offensive substances are not destroyed by metallic salts, but only fixed; they appear again—the sulphuretted hydrogen by the action of an acid, the ammonia like compounds by that of a powerful fixed alkali. The manganates and permanganates, on the other hand, destroy the smelling substances completely; containing, as they do, a large quantity of oxygen, the very agent which accomplishes all natural disinfection, they give rise to an actual process of combustion, in consequence of which the cause of the odour or putrefaction is permanently removed. They resemble, in this respect, the alkaline hypochlorites, such as hypochlorite of potash, soda, or lime (chloride of lime), the action of which is likewise permanent. The hypochlorites act with less energy and rapidity than the manganates, and are in this respect inferior; but they have an advantage over the latter by their evolving chlorine in the gaseous state, and destroying in this manner odorous and putrefactive substances which are diffused in the atmosphere. But as the chlorine evolved is frequently found objectionable by, and injurious to, patients, it would be important to ascertain whether the same effect could not be accomplished by exposing the contaminated air to the action of extended surfaces of solutions of the manganates and permanganates, either contained in shallow vessels, or diffused over sheets of wire gauze. The manganates and permanganates have, moreover, the advantage of possessing peculiar and strongly marked colours, whereby they are readily and safely distinguished from other compounds. In consequence of this marked colouration, accidents which have been frequently caused by the incautious and erroneous use of hypochlorites, or of metallic salts, are scarcely possible with the manganates and permanganates, which are, moreover, in themselves comparatively innoxious." In reference to this mode of disinfection we have to add, that Mr. Condy has sent to the Editor of the *Sanitary Review* some of the disinfecting fluid, for experimental purposes. The editor has the matter under observation, but his experiments are not sufficiently extended to allow of a report in the present number.

### SANITARY REPORTS.

Mr. Alfred Dickens, C.E., Superintending Inspector to the Board of Health, in a Report on Droitwich, in the county of Worcester, states:—

"That the rate of mortality within the borough of Wich, or Droitwich, in the county of Worcester, is very high. That excess of disease may be distinctly traced to the undrained condition of many of the houses, to deficient ventilation, and to the want of a proper water supply. That the highways are badly kept at a large annual cost. That the condition of the inhabitants would be materially improved,

"I. By a thorough system of public and private drainage. II. By a supply of wholesome water laid on, as far as possible, to every house, and by the abandonment of all those wells now in existence, the water from which is admitted by all to be impure. III. By the substitution of more decent accommodation, in lieu of the present filthy privies. IV. By improved surface paving to all the courts, alleys, and highways throughout the town. . . . .

"7. That the application of the Public Health Act to Droitwich is not only necessary at the present time, but it is calculated to be of inestimable service hereafter.

"Whereupon I have the honour of recommending that the Public Health Act, 1848, be forthwith applied to the said borough of Droitwich."

In a Report on Windhill, in the West Riding of York, Mr. Dickens proposed that the Public Health Act should not be extended. In a third Report on Leamington Priors, Mr. Dickens recommends that the parishes of Milverton, and a portion of the parish of Lillington, should be added to the Leamington district. And in a fourth Report, he recommends that the Public Health Act should be applied to the town and Cinque Port of Seaford, in Sussex.

---

### WORKHOUSES AND THE MEDICAL OFFICERS OF HEALTH.

Dr. Septimus Gibbon has forwarded us certain official communications relating to the inspection of Workhouses and Medical Officers of Health. The pith of the matter is, that Dr. Gibbon, in the discharge of his duties, was compelled to make application to the Poor Law Board for permission of right to enter the Workhouse of the Holborn Union, in order to enable him to report thereon as a public edifice. The answer of the Poor-Law Board is in opposition to Dr. Gibbon's very proper demand. The Secretary of the Poor-Law Board writes as follows, to the Clerk to the Board of Guardians.

"The Board, however, while they incline to think that a Workhouse is not a public edifice within the meaning of this section of the Act, do not find that the Act gives the Medical Officer of Health any right or authority to enter the Workhouse for any purpose without the permission of the Guardians of the Union ; and the Board are, therefore, of opinion that it is competent to the Guardians to decline to admit him into the Workhouse if they should think fit to do so."

Dr. Gibbon thinks this is a decision which should be made known to all Medical Officers of Health. Precisely. But is it not like all circumlocution decisions and concerns—one department upsetting another ? Yet there are people weak enough to think that the disease evils which afflict our country, are removable by intrusting their removal to the circumlocution representatives, rather than to the instinct, energy, and knowledge of the nation at large.

